# Delayed Traumatic Rupture of the Spleen in a Patient with Mantle Cell Non-Hodgkin Lymphoma after an In-Hospital Fall: A Fatal Case

**DOI:** 10.3390/diagnostics14121254

**Published:** 2024-06-14

**Authors:** Giuseppe Davide Albano, Stefania Zerbo, Mario Spanò, Nello Grassi, Emiliano Maresi, Ada Maria Florena, Antonina Argo

**Affiliations:** 1 Institute of Legal Medicine, Department of Health Promotion, Mother and Child Care, Internal Medicine and Medical Specialties, University of Palermo, Via del Vespro 129, 90100 Palermo, Italy; stefania.zerbo@unipa.it (S.Z.); spano.mario94@gmail.com (M.S.); antonella.argo@unipa.it (A.A.); 2 General Surgery Department, Department of Surgical, Oncological and Oral Sciences, University of Palermo, Via del Vespro 129, 90100 Palermo, Italy; nello.grassi@unipa.it; 3 Pathological Anatomy Department, Department of Health Promotion, Mother and Child Care, Internal Medicine and Medical Specialties, University of Palermo, Via del Vespro 129, 90100 Palermo, Italy; emiliano.maresi@unipa.it (E.M.); adamaria.florena@unipa.it (A.M.F.)

**Keywords:** hospital fall, delayed splenic rupture, non-Hodgkin lymphoma, autopsy, forensic diagnosis, malpractice, immunohistochemistry

## Abstract

Splenic rupture and hematoma are significant complications that can occur in patients with non-Hodgkin lymphoma (NHL). Understanding these associated complications is essential for optimal patient management and enhanced patient outcomes. Histopathological and immunohistochemical analyses are crucial in diagnosing NHL and assessing splenic involvement. In this study, a judicial autopsy had been requested by the Prosecutor’s Office for a malpractice claim due to a fall in the hospital. In the Emergency Department, a 72-year-old man fell from a gurney and reported sustaining a wound to his forehead. No other symptoms were reported. A face and brain CT scan showed no abnormalities. Nine days after discharge, the patient presented with abdominal pain. An abdominal CT revealed splenic rupture and hemoperitoneum. The patient underwent open splenectomy but showed signs of hemodynamic shock and subsequently died. The evidence from the autopsy allowed us to diagnose mantle cell non-Hodgkin lymphoma with spleen involvement, previously unknown. Histopathological and immunohistochemical analyses were performed to assess the diagnosis of splenic rupture and estimate its timing. The findings strongly suggest that the splenic rupture was associated with the patient’s fall and the pre-existing malignancy. This case highlights the importance of considering an underlying hematological malignancy when investigating delayed splenic rupture. An immunohistochemical study of spleen samples allowed the timing of splenic hematoma and rupture to be assessed, leading to the establishment of a causal relationship with trauma.

## 1. Introduction

Splenic rupture typically occurs after a direct traumatic injury to the abdomen. However, the majority of traumatic splenic ruptures are acute events; a minority of patients present with a two-stage splenic rupture that occurs days to weeks after the abdominal trauma [1]. According to Baudet, the period from trauma to splenic rupture can be 48 h or more and is characterized by a variable asymptomatic period [2]. In such cases, 50% of patients experience hemorrhage within 1 week after the trauma, 25% within 2 weeks, and 10% after more than 4 weeks. Patients who develop an early rupture of the spleen die at a rate of around 1%, while the mortality associated with delayed rupture is approximately 15% [3,4,5].

Splenic rupture can also develop in the absence of trauma, although it is an infrequent event. Several pathologies have been associated with splenic rupture in the absence of trauma, most often malignant hematological disorder, viral infection, or local inflammatory disorder [6].

Among the malignant hematological disorders involved in splenic rupture, non-Hodgkin lymphoma (NHL) (34%) and acute myeloid leukemia (34%) are most frequently reported, followed by chronic myeloid leukemia (18%) and lymphoblastic acute leukemia [7]. These occur due to possible splenic involvement in these disorders, which determines the congestion of the splenic parenchyma by blast cells. Therefore, according to the scientific literature, splenic rupture and hematoma are significant complications that can occur in patients with NHL [8]. In some cases, NHL can also cause splenomegaly, making the spleen more susceptible to rupture due to trauma, or even spontaneously [9,10]. Rupture can lead to intraperitoneal bleeding, forming a hematoma. Histopathological and immunohistochemical analyses are crucial in diagnosing NHL and assessing the extent of spleen involvement [10]. Identifying such complications is essential for timely intervention and optimal patient management. In this regard, previous research has underscored the significance of conducting histochemical and immunohistochemical analyses of subcapsular splenic hematomas. This has yielded valuable findings for determining the timeline of subcapsular hematoma development, in addition to the need to establish a causal link between splenic rupture and a preceding traumatic incident [11]. Understanding this association can enhance patient care and outcomes and help to establish a causal relationship in the forensic context. The aim of this report is to explore the histopathological and immunohistochemical aspects involved in diagnosing traumatic splenic rupture in a patient with mantle cell non-Hodgkin lymphoma who died 10 days after a fall in the hospital via a judicial autopsy for a medical malpractice case.

## 2. Case Report

A 72-year-old male was admitted to the hospital with a several-day history of asthenia and hypotension. The patient had a positive medical history for chronic atrial fibrillation and hypertension, in treatment with anticoagulant therapy (warfarin), and beta and alpha blockers. While he was at the Emergency Department (ED), he fell from a gurney and reported sustaining a lacerated, contused wound to his forehead. No other symptoms were reported, and a CT scan of the face and brain showed no abnormalities (Supplementary Materials). No significant clinical or blood test elements suggested urgency, and his vital signs were normal. Therefore, he was discharged. Nine days after discharge, the patient presented with abdominal pain, pallor, and dyspnea, and was taken to the emergency room. Laboratory tests after the second admission to the Emergency Department showed the following results: red blood cells 3.13 × 10^6^/μL, hemoglobin 8.8 g/dL, hematocrit 26.9%, platelets 158 × 10^3^/μL PT 32%, PTT 44 seconds, D-Dimer 4764 ng/mL, lactates 8.8 mmol/L.

An abdominal CT revealed splenomegaly, splenic rupture, and hemoperitoneum (Supplementary Materials). An emergent open splenectomy was performed, but the patient showed signs of shock and hemodynamic instability. After surgery, he was transferred to the intensive care unit; subsequently, death occurred as a consequence of hemorrhagic shock.

The prosecuting officer requested a judicial autopsy for a malpractice claim due to the fall in the ED and a lack of surveillance. All of the patient’s medical records were analyzed. At the time of the autopsy, the spleen, which had been removed during the life-saving operation, was examined macroscopically. All organs and tissue samples were routinely fixed in 10% neutral buffered formalin. Microscopic examination was carried out with different histochemical and immunohistochemical stainings.

We performed hematoxylin–eosin staining and estimated the histopathological timing of the subcapsular hematoma and hemorrhage with van Gieson staining and CD68 expression. Immunohistochemistry targeted multiple lymphoma markers for lymph nodes, spleen, and bone marrow samples, including CD20, CD5, CD3, CD10, Bcl-2, BCl-6, and Ki-67.

### 2.1. Autopsy Findings

At the time of the autopsy, the corpse appeared well preserved with no signs of putrefaction. The height was 170 cm and the weight was 100 kg. The corpse showed signs of laparotomy. Furthermore, on the forehead, there was a sutured, lacerated, contused wound measuring 2.5 × 0.5 cm.

**Figure 1:**
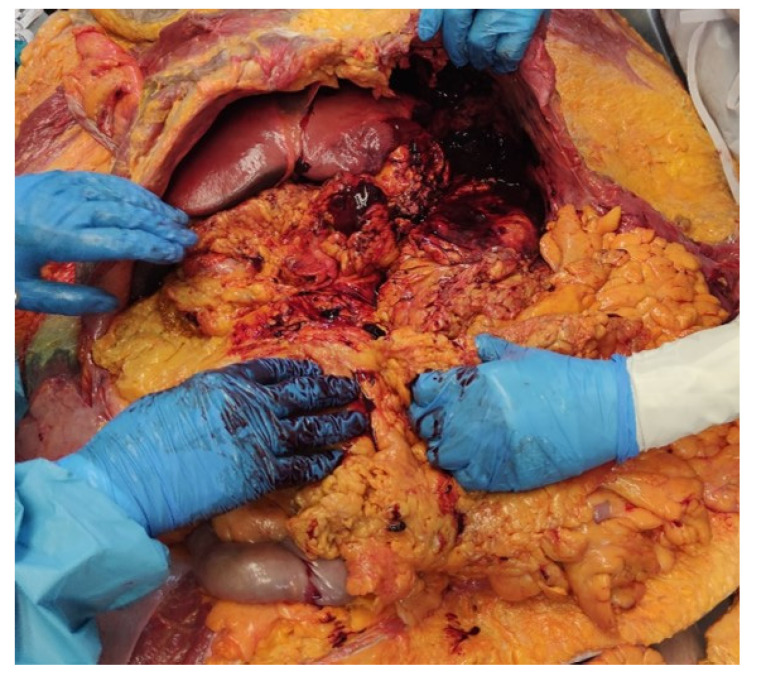
Blood clots in splenic lodge.

The autopsy revealed an increased volume of paratracheal and mesenteric lymph nodes, which were taken for histological investigation, and blood and blood clots in the splenic lodge (Figure 1). No other significant macroscopic findings were identified.

**Figure 2:**
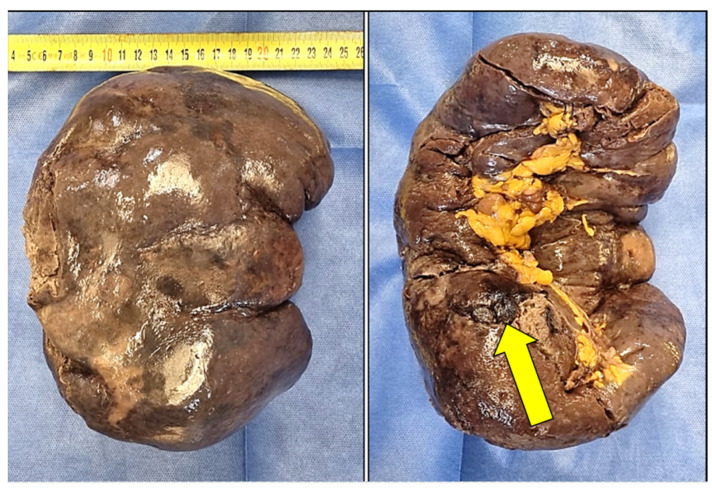
Spleen, macroscopic view. Yellow arrow highlights subcapsular hematoma in splenic hilum.

**Figure 3:**
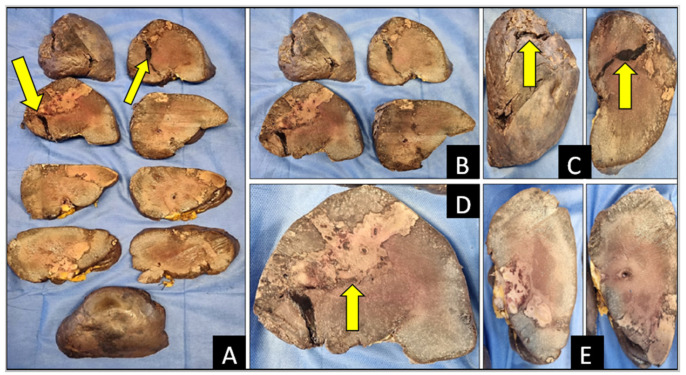
Transverse sections of spleen. Yellow arrows show areas of (**A**–**D**) infarction, and (**A**–**C**) hemorrhage and hemorrhagic dissection. Subcapsular infarction is visible also (**E**).

After fixation in formaldehyde solution, the spleen measured 24 × 12 × 18 cm (Figure 2) and weighed 413 g. Macroscopic examination showed severe splenomegaly with a subcapsular hematoma (Figure 2 and Figure 3). On the convex surface of the organ, there was a 5 cm capsular laceration (Figure 3). Along the convex surface, there were multiple grayish subcapsular infarcted areas. On the section surface (through cuts along the minor axis), there were areas of hemorrhagic dissection underlying the superficial laceration that extended almost to the total thickness of the organ (Figure 3). From the convex to the concave margin, there were grayish infarcted areas with a map-like appearance (Figure 3), ranging in size from 2 cm to 8 cm. There were multiple areas of hemorrhagic infarction in the subcapsular area, affecting the hilar surface and the upper pole of the organ.

### 2.2. Histological and Immunohistochemical Findings

**Figure 4:**
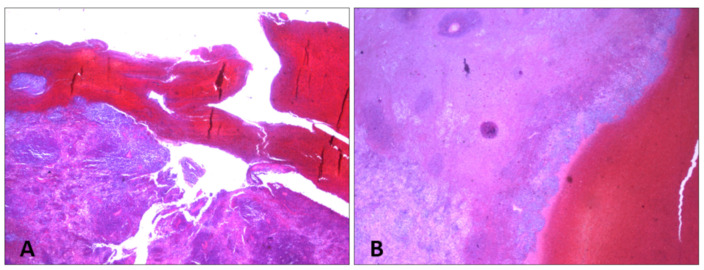
H&E staining of spleen samples. (**A**) Subcapsular hematoma is stratified under capsule, which appears fissured (10×). (**B**) Macrophages containing hemosiderin at interface between hematoma and parenchyma (10×).

**Figure 5:**
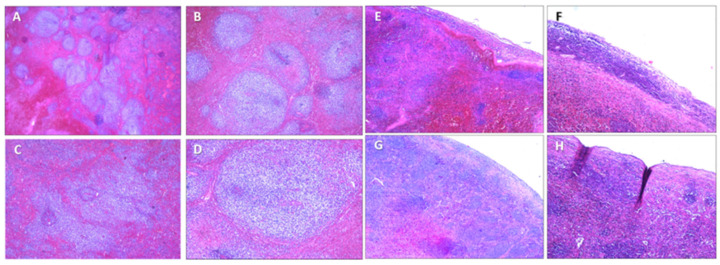
H&E staining of spleen samples. (**A**–**C**) Numerous neoplastic follicles next to each other (back to back) (10×). (**D**) Neoplastic follicle showing lack of polarization with random distribution of centrocytes and centroblasts, loss of defined mantle area, and absence of macrophages with dyeable body (10×). (**E**–**H**) Perisplenium-infiltrating mantle cell lymphoma with subcapsular hemorrhages (10×).

Microscopic examination with hematoxylin–eosin staining showed that the splenic tissue had a subverted structure caused by hemorrhagic phenomena, which partially dissected the parenchyma up to the perisplenium (Figure 4 and Figure 5) in a serpiginous manner. This was associated with an abundance of abnormal tumor cells, particularly a monomorphic lymphoid proliferation of small-to-medium-sized cells with nuclei with irregular outlines (Figure 4 and Figure 5). 

**Figure 6:**
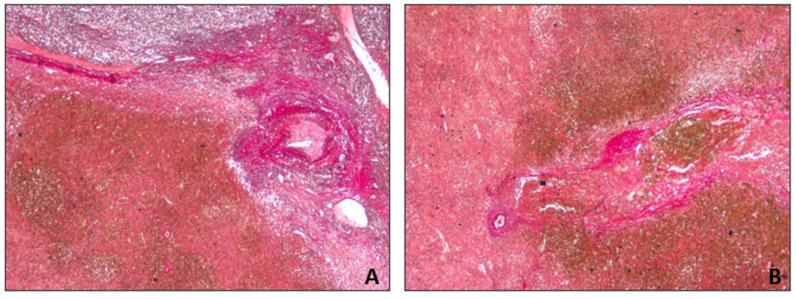
Spleen sections with van Gieson staining. Perivascular fibrosis and hemorrhage: (**A**) 10×; (**B**) 20×.

**Figure 7:**
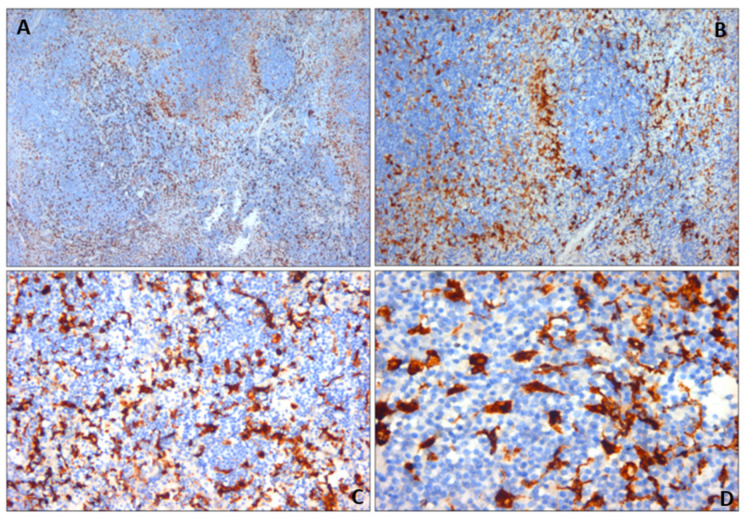
Spleen sections with CD68 immunohistochemical markers. Active histiocyte proliferation in splenic hematoma: (**A**) 10×; (**B**) 20×; (**C**) 40×; (**D**) 80×.

Estimation of the subcapsular hematoma and hemorrhage timing was carried out with H&E, van Gieson staining, and CD68 expression. The van Gieson staining highlighted perivascular fibrosis (Figure 6). The immunohistochemical analysis for CD68 revealed the presence of CD68-positive cells (Figure 7), specifically histiocytic macrophages.

**Figure 8:**
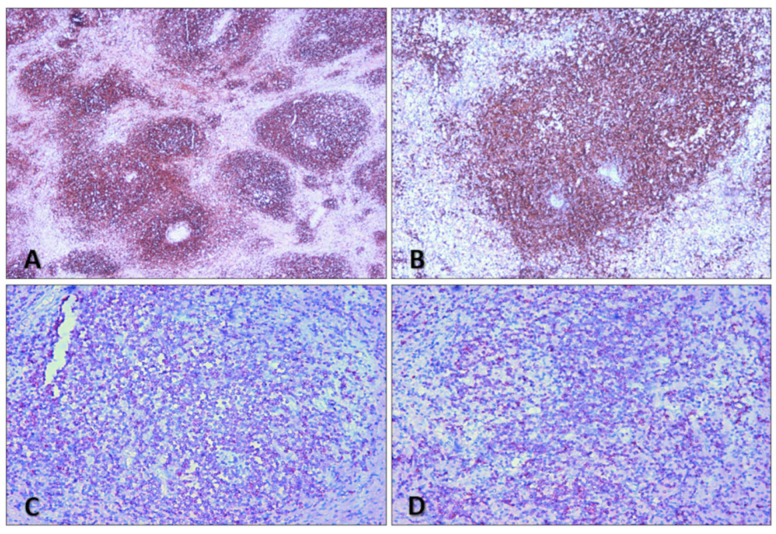
Spleen sections. (**A**,**B**) CD20 expression (10×, 20×). (**C**,**D**) Bcl-2 expression (40×). Mantle cell lymphoma features.

We conducted immunohistochemical investigations to characterize the spleen neoplasm. Immunohistochemistry showed the presence of CD20-, CD5-, and BCl-2-positive cells (Figure 8).

**Figure 9:**
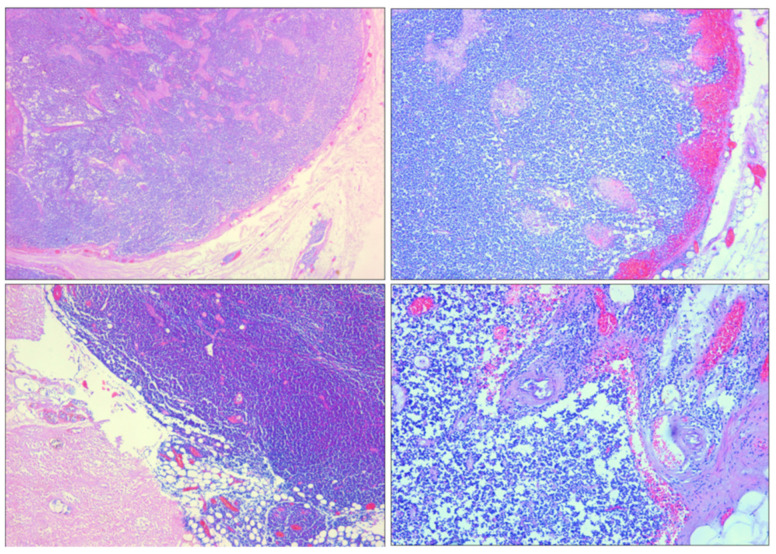
H&E staining of lymph node section—site of widespread lymphomatous cell infiltration (10×).

A microscopic examination of the mesenteric and paratracheal lymph nodes was also performed. Lymph nodes were sites of widespread lymphomatous cell infiltration with the same monomorphic lymphoid proliferation as the spleen. The immunohistochemical analysis showed that lymphoid cells were BCl-2+, CD5+, and CD20+, with a proliferation index (Ki67) of approximately 75% (Figure 9). 

**Figure 10:**
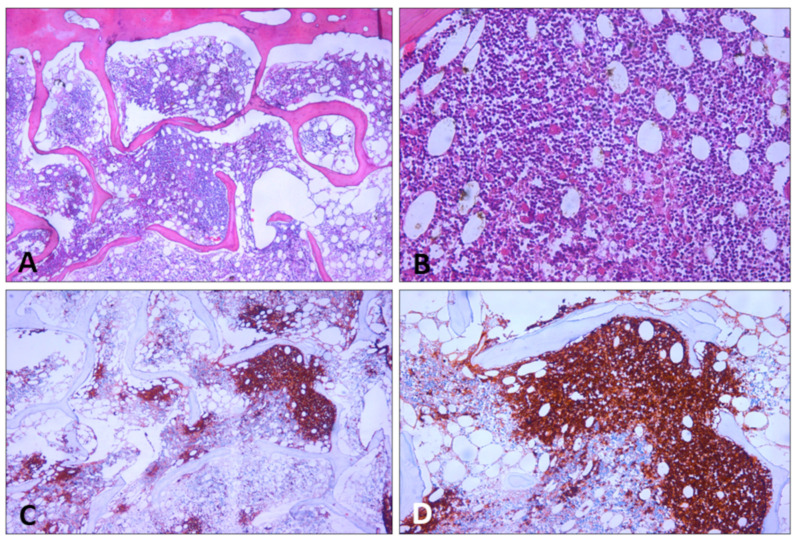
Bone marrow sections. (**A**,**B**) H&E staining. (**C**,**D**) CD20 expression. Hematoxylin–eosin stain and CD20 show paratrabecular and intramedullary nodular lymphoid infiltration ((**A**,**C**) 10×, (**B**,**D**) 20×).

The microscopic examination of the bone marrow with hematoxylin–eosin staining and CD20 showed paratrabecular and intramedullary nodular lymphoid infiltration (Figure 10).

## 3. Discussion

Mantle cell lymphoma is a rare type of B-cell non-Hodgkin lymphoma, representing less than 10% of NHL cases. It usually occurs in the fifth decade and shows a predominance in men. Mantle cell lymphoma is characterized by t (11;14) with CCND1-IGH fusion, leading to the overexpression of cyclin D1 (Bcl-1), a protein required for progression through the G1 phase of the cell cycle. It manifests as a spectrum of diseases, ranging from relatively indolent to aggressive. High levels of cyclin D1 characterize the immunophenotype. Most tumors also express CD19 and CD20. Tumors are generally CD5-positive and CD23-negative, which is a useful characteristic to distinguish it from chronic lymphocytic leukemia/small lymphocytic lymphoma (CLL/SLL) [12]. Tumor cells can surround the reactive germinal centers of the lymph nodes, resulting in a nodular appearance at low magnification, or they can subvert the typical architecture of the lymph nodes diffusely. Typically, proliferation consists of a homogeneous population of small lymphocytes with irregular outlines. Chromatin is often condensed, nucleoli are inconspicuous, and cytoplasm is sparse. Sometimes, neoplasms of intermediate-sized cells with more dispersed chromatin and a high mitotic index are observed. At the time of diagnosis, most patients show generalized lymphadenopathy. Extra lymph node involvement frequently includes the bone marrow, spleen, liver, and gastrointestinal tract. Based on the immunohistochemical analysis, the patient was diagnosed with mantle cell non-Hodgkin lymphoma, as evidenced by CD20-, CD5-, Bcl-2-, and Ki-67-positive (>75%) cells. According to the Ann Harbor classification, the evidence of splenic, medullary, and supra- and subdiaphragmatic lymph node involvement indicates that the neoplasm was stage IV A.

Van Gieson staining and CD68 were particularly useful in highlighting the timing of the subcapsular hematoma and necrotic infarction of the parenchyma [11]. The presence of perivascular fibrosis and histiocytic macrophages indicates that the repair process had already developed. Therefore, the splenic hematoma was compatible with a previous trauma, and then with a two-stage rupture. Moreover, the timing of the rupture was consistent with a delayed splenic rupture due to the traumatic fall (within approximately 10 days). The findings strongly suggest that the splenic rupture experienced after the trauma may have been associated with the patient’s pre-existing hematological splenic malignancy. The cause of death was attributed to cardiocirculatory arrest due to hypovolemic shock in a patient subjected to splenectomy for splenic rupture and severe hemoperitoneum due to an in-hospital fall, affected by mantle cell type B non-Hodgkin lymphoma (Ann Arbor stage IV A). The lymphoma diagnosis was made after death via autopsy. The lymphoproliferative disease was unknown prior to the autopsy.

This case highlights the importance of considering underlying hematological malignancies when investigating delayed traumatic splenic rupture. Histopathological and immunohistochemical analyses are crucial for establishing accurate forensic diagnoses, better understanding potential complications, and guiding appropriate patient management. The study of erythrocytes, fibrinogen, platelets, and macrophages offers valuable insights into the progression of reparative processes linked to subcapsular hematoma and subsequent delayed splenic rupture. Our research underscores the significance of the histochemical and immunohistochemical examination of subcapsular splenic hematomas, which can yield beneficial outcomes for determining the timeline of their formation. However, it is necessary to establish a causal connection between splenic rupture and a preceding traumatic event [11]. In this case, following the fall event, only a face and brain CT scan was performed. In fact, in the hours following the fall, there were no signs or symptoms indicating abdominal involvement. It was not known that the patient had splenic lymphoma. 

Medical malpractice claims fall within tort law, which addresses allegations arising from harm caused, typically grounded in negligence. This legal framework hinges on principles such as duty of care, breach of duty, resulting damage, and the establishment of a causal link between the violation and the harm incurred [13]. Many falls that occur in hospitals are not preventable [14]. In Italy, according to Italian legislation, clinical negligence can be claimed in cases where there is a lack of adherence to evidence-based guidelines and good clinical practices in daily activities [15]. No data on lack of care, lack of surveillance, or lack of preventive actions have emerged regarding the onset of falls. In the present case, criminal liability of the physicians for the death was excluded, given the absence of signs and symptoms indicating abdominal involvement and the absence of clinical negligence and lack of surveillance from the medical records analysis. Patients with lymphoproliferative disease with possible splenic localization should undergo more accurate clinical instrument monitoring in case of trauma, since the risk of rupture exists and is known in the literature [7,8,9,10].

As outlined previously, spontaneous splenic rupture typically does not occur due to trauma. The individual in this case was diagnosed with non-Hodgkin lymphoma only after death, and he may have experienced a spontaneous splenic rupture. Considering the history of the fall and the timing of the splenic rupture (almost 10 days later), according to the histological findings of the splenic samples (with a presence of fibrin and macrophages in the subcapsular hematoma), the rupture was considered to be most probably of traumatic origin, with an underlying contributing pathology.

The rib cage typically protects the spleen against trauma. However, in cases of splenic pathology, the spleen becomes more susceptible to rupture due to changes in its consistency and because splenomegaly can cause the organ to extend below the rib cage. Abdominal pain is typically the primary symptom of splenic rupture. The nature of this pain can vary, ranging from localized pain in the left upper quadrant to left-sided chest pain or generalized abdominal discomfort. Patients with splenic injuries can also experience symptoms such as nausea, vomiting, fainting, abdominal swelling, low blood pressure, rapid heartbeat, signs of peritoneal irritation, fever, anemia, and Kehr’s sign (pain in the left shoulder due to irritation of the diaphragm). It is essential to consider the possibility of splenic injury for all patients with hematologic malignancies, even in the absence of trauma. The presentation of splenic injury can differ between individuals with hematologic malignancies and those with healthy spleens. Furthermore, in patients for whom the situation is unknown, the history should be checked for any symptoms suggestive of lymphoma, since it may be a condition that predisposes patients to a two-stage rupture, an event marked by high mortality.

## 4. Conclusions

Hematological malignancies need to be considered as a contributing factor when investigating delayed traumatic splenic rupture. Histopathological and immunohistochemical analyses play a crucial role in diagnosing non-Hodgkin lymphoma and estimating the time of splenic rupture. Van Gieson staining and immunohistochemical analysis with CD68 markers are reliable methods to establish the time since the trauma of a subcapsular hematoma, an essential task in forensics when dealing with violent deaths and medical malpractice issues. Patients with a history of lymphoproliferative disease should undergo more accurate clinical instrument monitoring in case of trauma due to the existing risk of splenic rupture and mortality.

## Data Availability

The data presented in this study are available on request from the corresponding author.

## Supplementary Materials

The following supporting information can be downloaded at: https://www.mdpi.com/article/10.3390/diagnostics14121254/s1, Figure S1: Brain (A and B) and Bone (C) windows of Head CT performed after trauma. Absence of hemorrhages and bone traumatic lesions. Figure S2: Abdomen CT (abdomen window) performed after the second admission to Emergency department. Large subcapsular splenic hematoma with signs of hemorrhage in perihepatic and perisplenic regions.
