# Supplementary material for: Delayed Traumatic Rupture of the Spleen in a Patient with Mantle Cell Non-Hodgkin Lymphoma after an In-Hospital Fall: A Fatal Case
Source: Diagnostics (Basel). 2024 Jun 14;14(12):1254. doi: 10.3390/diagnostics14121254 (PMC11202632; doi:10.3390/diagnostics14121254)
Supplement: Supplementary file 1 [file diagnostics-14-01254-s001.zip › diagnostics-3042048-supplementary.pdf]

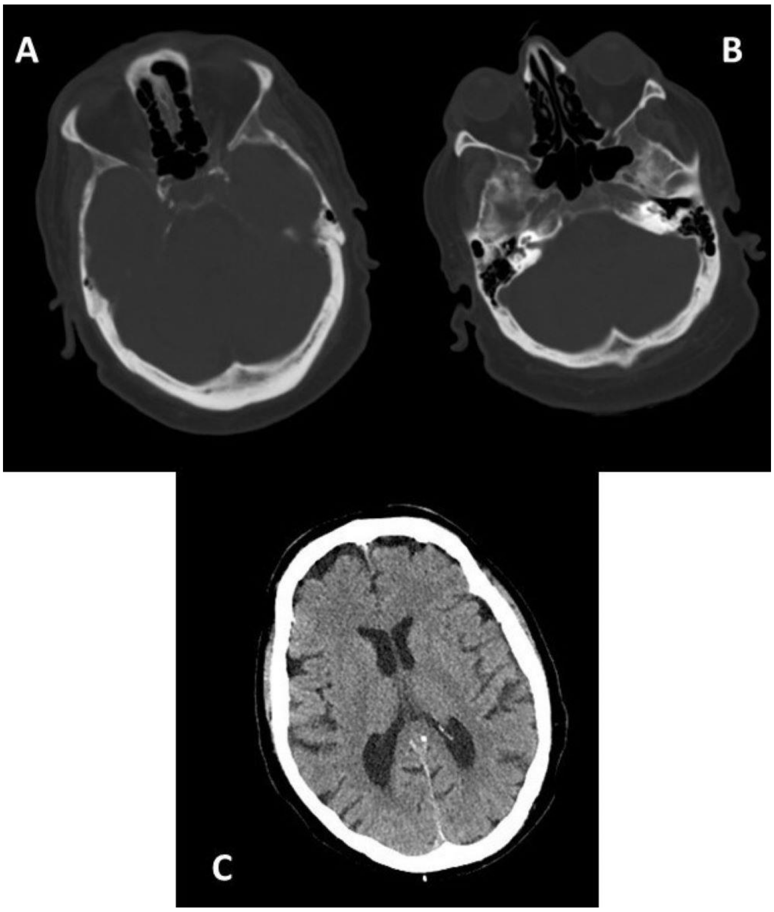

Figure S1. Brain (A and B) and Bone (C) windows of Head CT performed after trauma. Absence of hemorrhages and bone traumatic lesions.

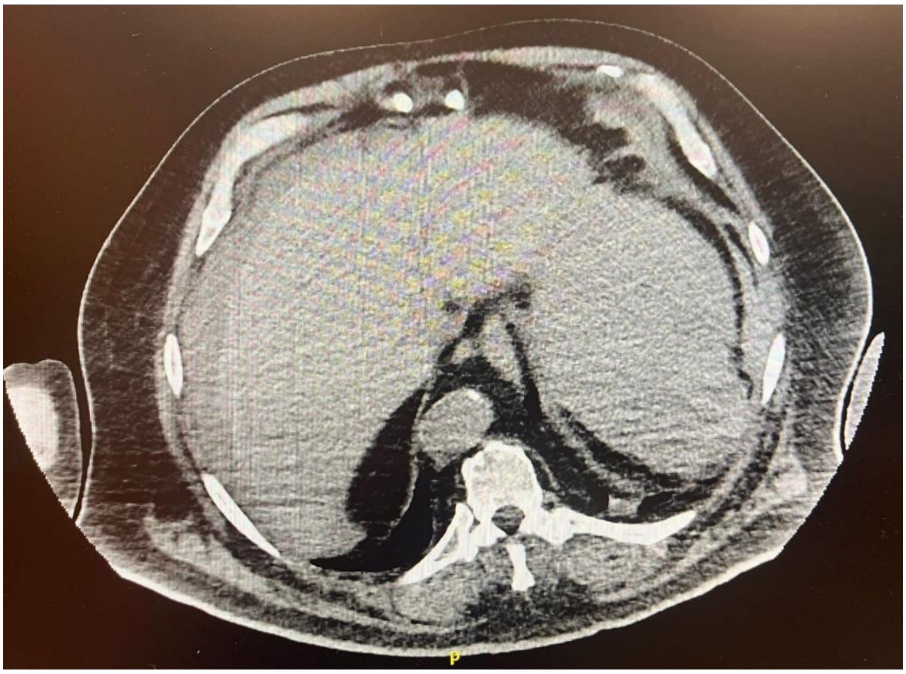

Figure S2. Abdomen CT (abdomen window) performed after the second admission to Emergency department. Large subcapsular splenic hematoma with signs of hemorrhage in perihepatic and perisplenic regions.
